# Spatial Match-Mismatch between Juvenile Fish and Prey Provides a Mechanism for Recruitment Variability across Contrasting Climate Conditions in the Eastern Bering Sea

**DOI:** 10.1371/journal.pone.0084526

**Published:** 2013-12-31

**Authors:** Elizabeth Calvert Siddon, Trond Kristiansen, Franz J. Mueter, Kirstin K. Holsman, Ron A. Heintz, Edward V. Farley

**Affiliations:** 1 School of Fisheries and Ocean Sciences, University of Alaska Fairbanks, Juneau, Alaska, United States of America; 2 Institute of Marine Research, Bergen, Norway; 3 Joint Institute for the Study of the Atmosphere and Ocean, University of Washington, Alaska Fisheries Science Center, National Marine Fisheries Service, National Oceanic and Atmospheric Administration, Seattle, Washington, United States of America; 4 Ted Stevens Marine Research Institute, Alaska Fisheries Science Center, National Marine Fisheries Service, National Oceanic and Atmospheric Administration, Juneau, Alaska, United States of America; University of Connecticut, United States of America

## Abstract

Understanding mechanisms behind variability in early life survival of marine fishes through modeling efforts can improve predictive capabilities for recruitment success under changing climate conditions. Walleye pollock (*Theragra chalcogramma*) support the largest single-species commercial fishery in the United States and represent an ecologically important component of the Bering Sea ecosystem. Variability in walleye pollock growth and survival is structured in part by climate-driven bottom-up control of zooplankton composition. We used two modeling approaches, informed by observations, to understand the roles of prey quality, prey composition, and water temperature on juvenile walleye pollock growth: (1) a bioenergetics model that included local predator and prey energy densities, and (2) an individual-based model that included a mechanistic feeding component dependent on larval development and behavior, local prey densities and size, and physical oceanographic conditions. Prey composition in late-summer shifted from predominantly smaller copepod species in the warmer 2005 season to larger species in the cooler 2010 season, reflecting differences in zooplankton composition between years. In 2010, the main prey of juvenile walleye pollock were more abundant, had greater biomass, and higher mean energy density, resulting in better growth conditions. Moreover, spatial patterns in prey composition and water temperature lead to areas of enhanced growth, or growth ‘hot spots’, for juvenile walleye pollock and survival may be enhanced when fish overlap with these areas. This study provides evidence that a spatial mismatch between juvenile walleye pollock and growth ‘hot spots’ in 2005 contributed to poor recruitment while a higher degree of overlap in 2010 resulted in improved recruitment. Our results indicate that climate-driven changes in prey quality and composition can impact growth of juvenile walleye pollock, potentially severely affecting recruitment variability.

## Introduction

The match-mismatch hypothesis [1] proposes that predator survival is dependent on the temporal and spatial overlap with prey resources [2]. Factors affecting temporal overlap, such as climate variability through altered phenology, can lead to changes in survival at critical life stages [3], [4]. Temporal variation in spatial patterns of physical or biological conditions may concurrently affect survival. For example, in temperate and sub-arctic marine ecosystems, the timing of the spring bloom varies between years, driven by physical oceanographic conditions that change due to climate variability (e.g., [5]). These conditions, such as the onset of stratification and light availability, also affect the spatial patterns of zooplankton abundance, which further influences the feeding success of planktivorous fish species. Hence, variability in the spatial overlap of predator and prey, as well as differences in prey quality [6], [7], may directly affect differences in year-class success of many marine fish species [8], [9].

Variability in year-class strength of gadids is often associated with changing physical conditions [10], [11]. The eastern Bering Sea (EBS) has experienced multi-year periods of both warm and cold conditions since the turn of the 21^st^ century [12], with cold years having much higher walleye pollock (*Theragra chalcogramma*) recruitment on average [13]. Changes in zooplankton composition between these periods have been identified as an important driver of recruitment success for walleye pollock [9], [14], but the mechanistic links remain poorly understood.

Interannual changes in ocean temperatures [12] and shifts in the spatio-temporal distribution of prey [14] make walleye pollock in the EBS an ideal case study to better understand drivers of recruitment success in sub-arctic marine fish. Larger zooplankton taxa, such as lipid-rich *Calanus* spp., were less abundant during recent warm years, possibly causing reduced growth rates and subsequent year-class strength of juvenile walleye pollock (hereafter juvenile pollock). In contrast, higher abundances of lipid-rich prey, combined with lower metabolic demands in cold years, may have allowed juvenile pollock to acquire greater lipid reserves by late summer and experience increased overwinter survival [9]. Although the energetic condition of juvenile pollock in late summer is recognized as a predictor of age-1 abundance during the following summer in the EBS [13], the causal mechanism linking differences in prey abundance and quality to walleye pollock survival remains untested.

The objectives of this study were to better understand the roles of prey quality, prey composition, and water temperature on juvenile pollock growth through (1) estimating spatial differences in maximum growth potential of juvenile pollock using a bioenergetics modeling approach, (2) comparing maximum growth potential to predicted growth from an individual-based model (IBM), and (3) quantifying the impact of prey quality, prey abundance, and water temperature on spatial variability in growth potential.

## Materials and Methods

### Ethics Statement

Collection of physical and biological oceanographic data and fish samples during the US Bering-Aleutian Salmon International Surveys (BASIS) conducted on the EBS shelf was approved through the National Marine Fisheries Service, Scientific Research Permit numbers 2005-9 and 2010-B1. Collection of biological data in the US Exclusive Economic Zone by federal scientists to support fishery research is granted by the Magnuson - Stevens Fishery Conservation and Management Act.

### Modeling Approaches

Two alternative modeling approaches were parameterized based on samples of juvenile pollock, zooplankton, and oceanographic data collected during the BASIS surveys conducted on the EBS shelf from mid-August to October 2005 and 2010 ([15]; Figure S1). We selected 2005 (warm) and 2010 (cold) for our analyses based on data availability and the pronounced contrast in ocean conditions between these years (e.g., depth-averaged temperature anomalies over the middle shelf; [12]). Extensive spatial coverage of the surveys, combined with varying climate conditions between years, provided ample data with which to inform the models and compare differences in predicted growth between a representative warm and cold year in the EBS.

Maximum growth potential from a Wisconsin-type bioenergetics model parameterized for juvenile pollock (modified from [16]) was compared with predicted growth from a mechanistic IBM [17]. Comparing model-based predictions of growth allowed for a better understanding of the mechanisms behind temporal and spatial variability in growth patterns and an evaluation of the importance of different model parameters. Growth (g⋅g^−1^⋅day^−1^; weight measures refer to wet weight throughout) was estimated for 65 mm standard length (SL; 2.5 g) juvenile pollock, corresponding to the average size of age-0 fish (<100 mm total length; TL) observed in late summer (2005: 64.1±6.7 mm SL [mean ± SD] and 1.97±0.93 g; 2010: 64.3±9.2 mm SL and 2.39±0.94 g; conversion between TL and SL followed [18]).

### Field Observations

#### Juvenile pollock abundance

Juvenile pollock were collected from the EBS shelf (inner domain: 0–50 m isobath, middle domain: 50–100 m isobath, and outer domain: 100–200 m isobath) using a midwater rope trawl following methods described in [19]. Catch per unit effort (CPUE; #⋅m^−2^) was calculated as:
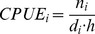
(1)where *n_i_* is the number of fish collected in a given haul *i*, *d_i_* is the trawl distance (m) calculated from starting and ending ship position, and *h* is the horizontal spread of the trawl net (m). Only surface tows at pre-defined stations were used to compute CPUE because midwater tows specifically targeted acoustic sign of walleye pollock.

#### Water temperature

Vertical profiles of water temperature were collected at each station sampled for oceanography using a Sea-Bird Electronics (SBE) conductivity-temperature-depth (CTD) profiler SBE-25 (2005) or SBE-911 (2010). The average temperature in the upper 30 m of the water column was used in the bioenergetics model, assuming juvenile pollock collected from surface trawls were concentrated within the upper 30 m [15]. For the IBM, the water column was divided into 1 m discrete depth bins. For all IBM simulations, the depth of the water column was set to the upper 100 m of all deeper stations (*n* = 9 of 116 in 2005, *n* = 27 of 160 in 2010) because MOCNESS data used to develop vertical profiles of zooplankton distribution (see ‘Zooplankton data’ below) was limited to 100 m. For stations with missing temperature data (*n* = 1 for 2005), data from the nearest station with similar depth was used. For stations with incomplete temperature profiles (*n* = 1 for 2005), temperatures were linearly interpolated between depths.

#### Zooplankton data

To characterize diets of juvenile pollock (<100 mm TL) across the EBS shelf in two contrasting years, samples collected from both surface (2005 and 2010) and midwater (2010) tows were used in the analysis (*n* = 26 stations in 2005, *n* = 47 stations in 2010 [*n* = 16 surface tows, *n* = 31 midwater tows]). Stomach content analyses followed standard methods as described in [19] to estimate the contribution of each prey taxon to the dietary volume of juvenile pollock (% volume). To compute overall average diet composition, contributions were weighted by the CPUE of juvenile pollock at each station and averaged across stations. All prey taxa of juvenile pollock that cumulatively accounted for at least 90% of the diet by volume and individually accounted for at least 2% of the diet by volume were included in the bioenergetics and IBM models (Table 1). Main prey taxa from either year were included in models for both years for comparing growth across years.

**Table 1:** Main prey taxa included in the models for 2005 and 2010.

2005			2010		
Taxa	Individual % Vol	Cumulative % Vol	Taxa	Individual % Vol	Cumulative % Vol
***Limacina helicina***	26.33		***Limacina helicina***	35.45	
*Pseudocalanus* sp.	26.04	52.4	*Thysanoessa inermis*	27.08	62.5
*Oikopleura* sp.	11.86	64.2	***Calanus marshallae***	13.87	76.4
*Centropages abdominalis*	8.98	73.2	*Neocalanus cristatus*	4.84	81.2
***Thysanoessa raschii***	8.48	81.7	*Thysanoessa inspinata*	3.16	84.4
*Thysanoessa* sp.	4.63	86.3	***Thysanoessa raschii***	3.09	87.5
*Acartia clausi*	3.40	89.7	*Neocalanus plumchrus* *	2.98	90.5
***Calanus marshallae***	1.71	91.4	*Eucalanus bungii*	2.95	93.4

Prey items cumulatively accounting for at least 90% of the diet by % volume and individually accounting for at least 2% of the diet by % volume were included. Prey taxa common to both years are shown in **bold**.

*Neocalanus plumchrus* was not identified in the 2010 bongo data, but did occur in the Juday data (small-mesh; not quantitative for large zooplankton taxa). Due to the absence in the bongo data, *N. plumchrus* was excluded from further analyses.

Water-column abundances of small and large zooplankton taxa were estimated from Juday and bongo net samples, respectively, as described in [14]. Small zooplankton representing main prey taxa included *Acartia clausi*, *Acartia* spp. (2010 only), *Centropages abdominalis*, and *Pseudocalanus* sp. Large zooplankton included *Calanus marshallae, Eucalanus bungii, Limacina helicina*, *Neocalanus cristatus, N. plumchrus* (2005 only), *Oikopleura* sp., *Thysanoessa inermis, T. inspinata*, and *T. raschii*.

Total sample weights (g) of taxa collected from the Juday net were computed from wet weight tables [20]. Densities (g⋅m^−3^) of taxa collected from the bongo net were measured during sample processing at the University of Alaska Fairbanks (2005; [21]) and NOAA/NMFS/Alaska Fisheries Science Center (2010). The year-specific average biomass of individuals for the main prey taxa was calculated by dividing the sum of the biomass of all specimens weighed (i.e., subsample) by the total number of specimens subsampled in a given year (Table S1).

Taxa-specific energy density (ED; kJ⋅g^−1^) values obtained from available zooplankton collections from the EBS during 2004 (warm; no ED data available from 2005) and 2010 (cold) were used to estimate average ED values during warm and cold conditions for the main prey taxa. For five taxa lacking sufficient information to estimate separate ED values, a single estimate was used in both years (Table S1). In these cases, only differences in abundance and biomass contributed to differences in average prey energy between years in the models. A biomass-weighted mean prey ED was calculated for each station and used as input to the bioenergetics model. At each station, the biomass of individual taxa was divided by the total prey biomass, multiplied by the taxa-specific energy density for each year, and summed across all taxa present at a given station.

Estimates of ED and % lipid were available for several copepod species (*C. marshallae*, *N. cristatus*, and *N. plumchrus*/*flemingeri*) from 2010 (see [22] for details on the biochemical processing). A linear regression was developed to predict species specific ED (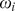) from % lipid values for other copepod species and/or climate conditions (Table S1), such that:
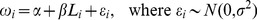
(2)where *α* and *β* represent the intercept and slope of the regression, respectively, *L_i_* is the lipid composition (%) of the individual copepod sample *i* and *ε_i_* is a residual. The residuals, *ε_i_*, are assumed to be independent and normally distributed with mean 0 and variance σ^2^ (*α = *19.3, p = 0.02; *β* = 0.41, p = 0.07; R^2^ = 0.98).

To account for diel vertical migrations, taxa-specific vertical profiles for day and night were developed for all main prey taxa as input for the IBM. Vertical profiles were based on summer MOCNESS surveys that provided depth-stratified abundance estimates. MOCNESS data were available for 2004 (warm) and 2009 (cold); these vertical profiles were applied to late-summer model runs for 2005 and 2010, respectively, assuming that the vertical behavior of zooplankton taxa is conserved seasonally and across years within similar oceanographic conditions. To assess the effect of this assumption, a sensitivity analysis was conducted using constant abundances by depth (see ‘IBM sensitivity analyses’ below).

In 2004, vertically stratified MOCNESS samples were collected at 5 daytime and 42 nighttime stations over the EBS shelf [21]. In 2009, 7 daytime and 22 nighttime stations were sampled (A. Pinchuk, unpubl. data). Daytime extended from approximately 07∶00 (sunrise) to 23∶30 (sunset) Alaska Daylight Savings Time during the sampling periods; stations sampled during crepuscular periods were excluded from the analysis. The depth increments of the MOCNESS varied depending on water depth; therefore, data were binned to the finest resolution available (i.e., 5–20 m increments). Zooplankton abundance was assumed to be uniform within sampling depths and averaged across all daytime and nighttime tows within a given year to obtain four vertical profiles for each taxon (day vs. night, 2004 vs. 2009). *Centropages abdominalis* were not collected by the MOCNESS and a uniform distribution throughout the water column was applied for both years because their distribution during the 2005 and 2010 BASIS surveys was predominantly at shallow, well-mixed stations of the inner domain. *Oikopleura* sp. did not occur in daytime tows in 2004; therefore, the 2009 daytime vertical distribution was applied for both 2005 and 2010 model runs. *Thysanoessa inspinata* were rarely collected by the MOCNESS (*n* = 1 for 2005; *n* = 3 for 2010), therefore an average vertical profile based on all *Thysanoessa* spp. was applied.

### Bioenergetics Model

A bioenergetics model was used to estimate spatially explicit maximum growth potential of juvenile pollock. We used the broadly applied Wisconsin bioenergetics modeling approach [23], [24] that has been adapted and appropriately validated for walleye pollock ([16], [25]; Table S2). The model estimates temperature- and weight-specific maximum daily (*d*) consumption for an individual fish at station *k* in year *t* (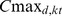 *; g*⋅*g* ^−1^⋅*d* ^−1^) as:
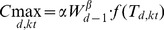
(3)where 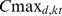 is parameterized from independent laboratory observations of consumption rates for the species, absent competitor or predator interference, and is assumed to scale exponentially with fish weight (*W)* according to α and β (the allometric intercept and slope of consumption) and thermal experience according to the temperature scaling function *f*(*T*) (Table S2).

Realized individual daily consumption rates (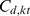; *g⋅g* ^−1^⋅d^−1^) based on *in situ* fish are typically much lower than 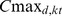 because inter- and intra-species competition, mismatched prey phenology or distributions, and predator avoidance behaviors by prey species often limit capture and consumption rates [16], [26]. The ratio of realized consumption to maximum consumption (i.e., 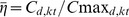), or the mean relative foraging rate, is a measure of *in situ* foraging efficiency. The rate 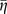 can be estimated using field observations of growth or it can be set to a specific value and used to predict daily growth (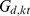) using the mass balance equation where growth is the difference between energy consumed (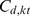) and energy lost to metabolism and waste (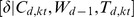), such that:
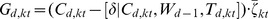
(4)where 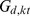 is the estimated daily specific growth (*g*⋅*g* ^−1^⋅*d* ^−1^), 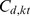 is realized consumption (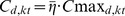), 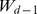 is the weight of an individual fish at the start of the simulation day *d*, 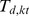 is the water temperature on simulation day *d*, and 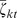 is the ratio of predator energy to prey energy density and is used to convert consumed biomass of prey into predator biomass (for more information see [26]). We used station-specific energy densities for prey (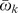) but annual mean predator energy density (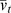) for year *t* because predator information was not available at all stations.

Energy density values for the main prey taxa were used to derive mean station-specific (*k*) available prey energy density for both years (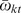); diet composition was assumed to be proportional to the relative biomass of zooplankton prey at each station. Individual fish energy density (*v_i_*) was determined using biochemical processing (see [22]). At stations where sufficient numbers of juvenile pollock were collected (*n* = 91 in 2005 and *n* = 12 in 2010), 2–8 fish were selected to represent the size range at each station. Station-specific mean energy density in a given year (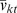) was weighted by CPUE and the number of fish processed at each station to calculate the average fish energy density by year (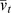).

We ran the model for a single simulation day (i.e., *d* = 1) using base scenario input parameter values (Table 2; see also [16] Tables I and II) that were kept constant across stations and years (i.e., *W*_ = _2.5 and 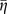 = 1), were constant across stations but varied by year (i.e., 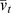), or varied by station and year (i.e., *T_kt_* and 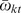). Because the model is size-specific, running the model for a single simulation day minimized compound errors that can accumulate over multiple simulation days when predicting growth and allowed for a comparative index of growth across stations. Keeping fish starting weights (*W*) constant allowed us to evaluate spatial effects of changes in the other parameters; setting η = 1 implies that growth was constrained by physiological processes, but not by prey consumption, hence we evaluated variability in maximum growth potential. Annual average fish energy density was applied across stations in each year (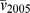 = 3.92 kJ⋅g^−1^; 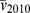 = 5.29 kJ⋅g^−1^).

**Table 2:** Parameter definitions and values used in the bioenergetics model to estimate maximum growth potential (*g*⋅*g* ^−1^⋅*d* ^−1^) of juvenile walleye pollock.

Parameter	Definition (units)	Value	Reference
*C*	Consumption (*g*⋅*g* ^−1^⋅*d* ^−1^)		
*η*	Relative foraging rate	0–1	*a*
O_2_ cal	Activity multiplier; convert g O_2_ → g prey	13560	*a*
*α*	Intercept of the allometric function for *C*	0.119	*a*
*β*	Slope of the allometric function for *C*	−0.46	*a*
*Q_c_*	Temperature dependent coefficient	2.6	*b*
*T_co_*	Optimum temperature for consumption	10	*b*
*T_cm_*	Maximum temperature for consumption	15	*b*
*R*	Respiration (g O_2_⋅g^−1^⋅day^−1^)		
*A_r_*	Intercept of the allometric function for *R*	0.0075	*b*
*B_r_*	Slope of the allometric function for *R*	−0.251	*b*
*Q_r_*	Temperature dependent coefficient	2.6	*b*
*T_ro_*	Optimum temperature for respiration	13	*b*
*T_rm_*	Maximum temperature for respiration	18	*b*
*D_s_*	Proportion of assimilated energy lost to Specific Dynamic Action	0.125	*b*
*A_m_*	Multiplier for active metabolism	2	*b*
*F*	Egestion		
*F_a_*	Proportion of consumed energy	0.15	*b*
*U*	Excretion		
*U_a_*	Proportion of assimilated energy	0.11	*b*

Parameters were used as inputs to the bioenergetics model described in [16].

^a^ [25]; *^b^* [16].

#### Bioenergetics sensitivity analyses

Individual input parameters were increased and decreased by 1 standard deviation (SD) and the change in growth relative to maximum predicted growth under the base scenario was recorded. A pooled SD was calculated across stations after removing the annual means. Relative foraging rate (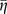) was held at 1 for all sensitivity model runs in order to compare the relative effect of other parameters on maximum growth potential.

Station-specific parameters (i.e., *T_kt_* and 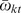) were varied to evaluate the relative effect on predicted growth and to examine resulting changes in spatially explicit growth patterns in each year. To evaluate the effect of variability in fish starting weight and energy density (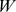 and 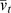, respectively) on estimated growth in 2005 and 2010, we used Monte Carlo simulations at a representative station (see Figure S1). A single station was used because mean fish weight and energy density input values did not vary across stations in the model due to data limitations; hence the spatial pattern in estimated growth is not affected by varying these values by a constant amount. The model was run 1000 times using parameter values drawn at random from a normal distribution with the observed mean and SD for each parameter. The resulting distribution of predicted maximum growth potential was examined.

### Mechanistic Individual-based Model

A mechanistic, depth-stratified IBM was used to predict average growth (*g*⋅*g* ^−1^⋅*d* ^−1^) and depth (m) of 100 simulated juvenile pollock by station. The details of the IBM and model validation are described in [17], [27]. The IBM was reparameterized for juvenile pollock and forced with input data for water column temperatures and prey availability in 1 m discrete depth bins. Prey abundance (#⋅m^−3^) was allocated into depth bins according to vertical profiles of zooplankton distribution and scaled to station depth.

The IBM used a mechanistic prey selection component that simulated the feeding behavior of juvenile pollock on zooplankton. The species composition of main prey taxa was based on observations; stage-specific length and width estimates were based on literature values or voucher collections from the EBS (Table S1). Optimal prey size was estimated to be 5–8% of fish length based on larval Atlantic cod research [28], [29]; juvenile pollock are predicted to have nearly 100% capture success for prey smaller than 5% of fish length, while the probability of capture success decreases with larger prey [17]. The simulated feeding ecology depended on juvenile pollock development (e.g., swimming speed, gape width, eye sensitivity) and vertical migratory behavior, prey densities and size, as well as light and physical oceanographic conditions (for details see [17]). Gape width was calculated as a function of fish size; conversion between length and weight followed [30]. Juvenile feeding processes were modeled with light-dependent prey encounter rates and prey-capture success (see [28]).

Vertical migratory behavior was modeled assuming that juvenile pollock would seek deeper depths to avoid visual predation risk as long as ingestion rates would sustain metabolism and growth. If not, juvenile fish would seek the euphotic zone where light enhances feeding success, but also increases predation risk. Prey distributions switched between daytime to nighttime profiles when the light level (i.e., irradiance) reached 1 µmol⋅m^−2^⋅s^−1^ [27]. The cost of vertical migration was included as a maximum of 10% of standard metabolic rates if the fish swims up or down at its maximum velocity, and scaled proportionally for shorter vertical displacements. Swimming velocity was a function of juvenile fish size [31].

Gut fullness was estimated based on the amount of prey biomass that was ingested and digested per time step (1 hour) according to the feeding module. Prey biomass flowing through the alimentary system supplied growth up to a maximum growth potential (*C_ma_*_x_; [25]), and standard metabolic cost, egestion, excretion, and specific dynamic action [16] were subtracted. Both maximum growth and metabolic costs were functions of fish weight and water temperature.

For all base model scenarios, the starting weight of the fish was held constant across stations, while zooplankton abundance and vertical distribution varied according to observations. Fish starting weight was 2.5 g ±30% assuming a random uniform distribution around the mean. Year-specific vertical profiles (day and night) for the main prey taxa and station-specific temperature and prey abundance profiles were applied. The model scenarios were run for 72 hours, but only the last 24 hours of the simulations were used for the analysis to avoid the early part of the simulations that may be unduly influenced by random initial conditions.

#### IBM sensitivity analyses

Fish starting weights and the vertical prey profiles were varied and resulting growth and average depth predictions were compared to values under the base model scenario (see [27] for sensitivity of the IBM model to variability in other parameters). To evaluate the effect of fish size separately from the effects of environmental controls, estimated growth based on fish starting weights of 2.0 g ±30% was compared to the base scenario (2.5 g ±30%), encompassing the mean weight of juvenile pollock from the BASIS surveys in 2005 (1.97±0.93 g, mean ± SD) and 2010 (2.39±0.94 g, mean ± SD). To test the effect of vertical distributions and diel migrations of prey taxa, model runs assuming a uniform distribution of prey with depth were compared to the base scenario, highlighting the effects of non-uniform zooplankton distribution and diel vertical migrations on juvenile pollock prey selection.

## Results

### Field Observations

#### Juvenile pollock abundance

**Figure 1:**
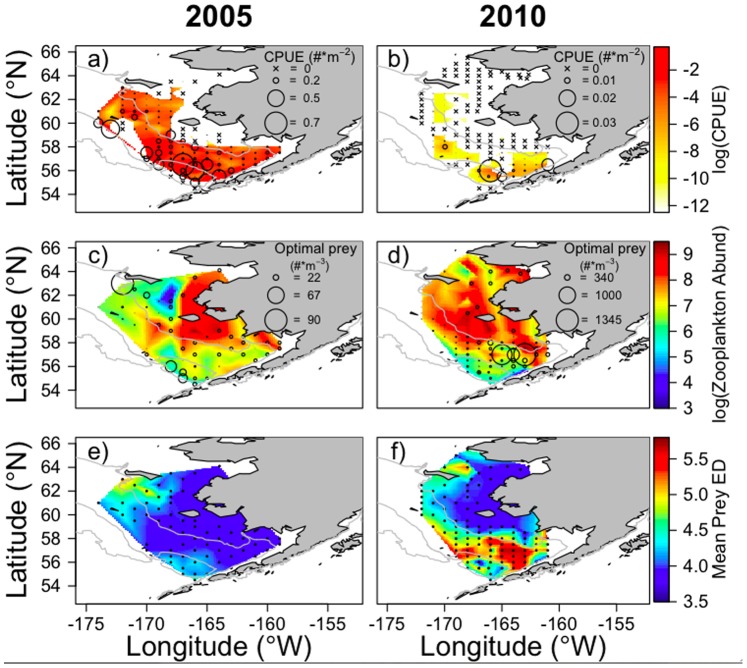
Log(CPUE) of juvenile walleye pollock collected in surface trawls in 2005 (a) and 2010 (b). Circle size is proportional to catch (#⋅m^−2^) at each station; note difference in scale between years. Stations with zero catch (×) are shown on white background. Log of total zooplankton abundance (#⋅m^−3^) for the main prey taxa is shown for 2005 (c) and 2010 (d). Circle size is proportional to the abundance of zooplankton within the optimal size range for 65 mm SL juvenile pollock (5–8% of fish length); note difference in scale between years. Biomass-weighted mean energy density (ED) of available zooplankton prey is shown for 2005 (e) and 2010 (f).

Juvenile pollock abundance and distribution had distinct spatial patterns in the surface layer between warm and cold years, with a more northerly distribution in warm years. Specifically, during warm late-summer conditions of 2005 juvenile pollock were distributed over a broad extent of the middle and outer domain, while in the cooler late summer of 2010 fish were concentrated over small regions of the southern shelf and outer domain (Figure 1, a and b). Abundance also varied between years with higher mean CPUE observed in 2005 as compared to 2010 (CPUE = 0.08 fish⋅m^−2^ vs. 0.001 fish⋅m^−2^, respectively) at positive catch stations.

#### Water temperature

The average water temperature in the upper 30 m of the water column during the BASIS survey was 8.8°C in 2005 and 7.6°C in 2010 (Figure S2, a and b), while the average temperature below 40 m was 4.5°C in 2005 and 2.9°C in 2010 (Figure S2, c and d). The warmest surface temperatures occurred in nearshore waters, although 2005 had warm temperatures over much of the southern shelf. Bottom temperatures reflected the extent of the cold pool (waters <2°C), which was limited to the northern portion of the study area in 2005 and covered much of the shelf in 2010.

#### Zooplankton

Diets of juvenile pollock shifted from smaller copepod species in the warmer 2005 summer season (e.g., *Pseudocalanus* sp.) to larger species in the cooler 2010 summer season (e.g., *N. cristatus*). Several large zooplankton species were present in the diets across years, including *L. helicina,* which was the predominant prey item in both years, as well as *C. marshallae* and *T. raschii*. In 2010, the main prey taxa of juvenile pollock collected in surface tows were similar to those from midwater tows, with the exception of *E. bungii* accounting for 0% and 3% of surface and midwater tows, respectively. *Eucalanus bungii* was included in further analyses because it represented approximately 3% of combined diets by volume (Table 1).

Changes in juvenile pollock diet composition reflect spatial and temporal variability in zooplankton species composition and availability. In 2005, the abundance of available prey was highest in the inner domain and decreased towards the outer domain and northern Bering Sea. The abundance of prey in 2010 was greater in the inner domain; in the southern region of the shelf abundances decreased towards the middle and outer domains (Figure 1, c and d). The lowest abundance of zooplankton occurred in areas corresponding to higher concentrations of juvenile pollock. The total abundance of zooplankton within the optimal prey size range for 65 mm SL juvenile pollock (species with mean length within 5–8% of fish length) was higher in the northwest region of the study area and over the southern shelf in the outer domain in 2005, with lesser overlap with juvenile pollock. In 2010, optimal prey was located across the middle and outer domains with highest abundances in the southern region, mirroring the distribution of juvenile pollock (Figure 1, c and d). Spatial patterns of zooplankton abundance accounting for all taxa <8% of fish length (not shown) reflected total abundance patterns in both years, indicating that areas of highest zooplankton abundance are driven by small (<5% of fish length) zooplankton taxa.

In 2005, available prey energy was highest in the northwest region of the shelf, with low prey energy over most of the shelf south of 60°N (Figure 1e) where juvenile pollock abundances were higher. In contrast, prey energy was very high across much of the southern shelf in 2010 (Figure 1f), particularly within the cold pool, where juvenile pollock were more abundant. Spatial patterns in prey energy were similar to spatial patterns of abundance for optimal prey size classes (Figure 1, c and d) because highest energy prey taxa are within 5–8% of fish length.

### Bioenergetics Model

**Figure 2:**
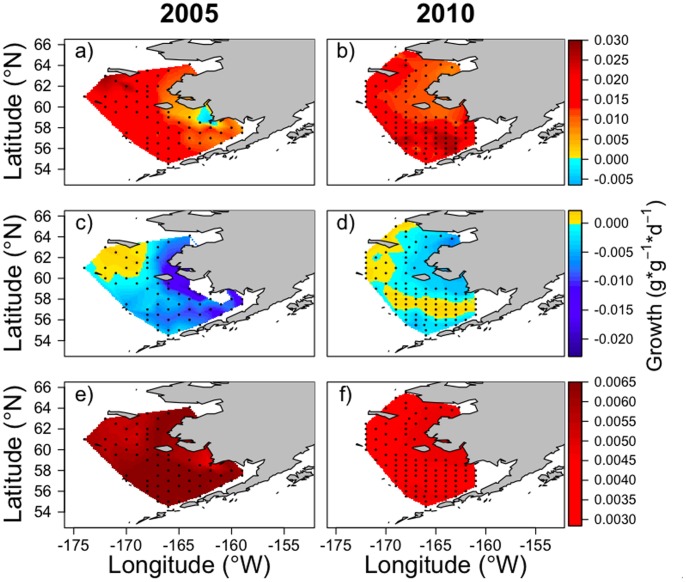
Predicted growth (*g⋅g* ^−1^⋅d^−1^) of juvenile walleye pollock from the bioenergetics model. Top panel (a and b) shows growth under the base model scenarios for 2005 and 2010 (*W* = 2.5 g, Temp = average temperature in upper 30 m, 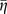 = 1.0, 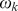 = prey energy density, 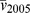 = 3.92 kJ⋅g^−1^; 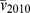 = 5.29 kJ⋅g^−1^). Middle panel (c and d) shows changes in predicted growth when temperature is increased by 1 standard deviation (SD). Predicted growth could not be estimated at one station in 2005 (c) in the inner domain under increased temperatures because the water temperature in the upper 30 m was greater than 15°C (*T_cm_* = 15°C in the model). Lower panel (e and f) shows changes in predicted growth when prey energy density is increased by 1 SD. Spatial plots of predicted growth when parameters are decreased by 1 SD are not shown, but can be visualized by subtracting the anomalies (lower two panels) from the base scenario plots (top panel).

Differences in the spatial pattern of maximum growth potential (*g*⋅*g* ^−1^⋅*d* ^−1^) of juvenile pollock occurred between a warm and cold year in the EBS (Figure 2, a and b). In 2005, growth potential was highest in the northwest region of the shelf (north of 60°N) and lowest in the inner domain with one station having negative growth. Gradients in growth potential, from low to high, occurred from the inner to outer domains and from southern to northern regions of the shelf (Figure 2a). In 2010, growth was positive at all stations with highest growth potential over the southern shelf and lower growth predicted in the northeast region (Figure 2b).

#### Bioenergetics sensitivity analyses

In 2005, increasing temperatures by 1 SD (Figure 2c) resulted in areas of decreased predicted growth at shallow inner domain and southern shelf stations where water temperatures already approached thermal thresholds. Growth could not be estimated at one inner domain station because the increased temperature exceeded 15°C, the maximum temperature for consumption (*T_cm_*) in the model. Decreasing water temperatures, resulting in increased growth, had the greatest effect in the same areas (not shown) because temperature-dependent control of growth is magnified where temperatures are close to thermal thresholds. In 2010, the effect of increasing water temperatures was an order of magnitude less than in 2005 (Table 3), but the spatial patterns were similar with shallow stations in the inner domain being most sensitive, as well as a small area in the outer domain (Figure 2d). Increasing available prey energy resulted in increased predicted growth rates across the region in 2005 (Figure 2e), with weaker effects in the inner domain and northwest region. In 2010, increased prey energy also resulted in elevated growth rates, but the magnitude of change was much lower than in 2005 and the spatial pattern differed; stronger effects occurred in the inner domain and southern region of the outer domain (Figure 2f).

**Table 3:** Summary of sensitivity analyses for the bioenergetics model in 2005 and 2010 showing the minimum (min), mean, and maximum (max) growth potential over all stations.

		2005			2010		
Parameter	SD	min	mean	max	min	mean	max
Base		−0.0056	0.0146	0.0291	0.0069	0.0172	0.0272
*W* +1 SD	0.935	−0.0056	−0.0041	−0.0017	−0.0052	−0.0037	−0.0023
*W* –1 SD	0.935	0.0034	0.0076	0.0103	0.0041	0.0068	0.0094
Temp +1 SD	1.75	−0.0227	−0.0053	0.0017	−0.0071	−0.0007	0.0018
Temp –1 SD	1.75	−0.0028	0.0018	0.0129	−0.0026	0.0008	0.003
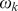 +1 SD	497.5	0.0046	0.0061	0.0065	0.0032	0.0044	0.0048
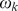 –1 SD	497.5	−0.0065	−0.0061	−0.0046	−0.0048	−0.0044	−0.0032
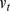 +1 SD	395.93	−0.0027	−0.0013	0.0005	−0.0019	−0.0012	−0.0005
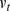 –1 SD	395.93	−0.0006	0.0016	0.0033	0.0006	0.0014	0.0022

Base values are predicted maximum growth potential (*g⋅g* ^−1^⋅*d* ^−1^) of juvenile pollock from the base model scenarios (*W* = 2.5 g, Temp = average temperature in upper 30 m, 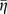 = 1.0, 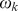 = prey energy density, 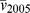 = 3.92 kJ⋅g^−1^; 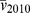 = 5.29 kJ⋅g^−1^). All other values denote the change in growth rate resulting from indicated changes in inputs; therefore (−) effects indicate that varied conditions resulted in lower predicted growth and vice versa. Pooled standard deviations (SDs) for each parameter were calculated across stations after removing the annual means. *W* and 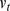 are constant values applied across all station, so changes (±1 SD) act as a scalar and result in similar spatial patterns across the area. Temperature and 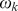 vary across stations.

**Figure 3:**
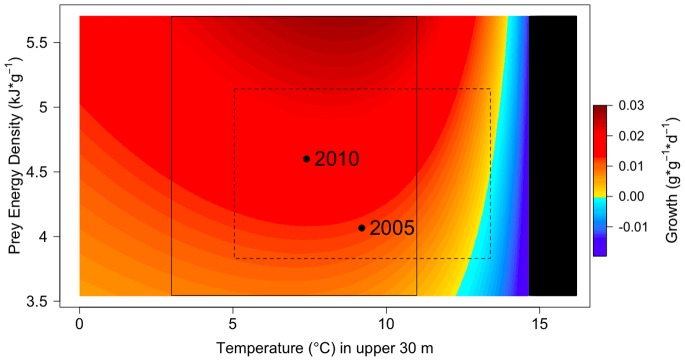
Predicted growth (*g*⋅*g* ^−1^⋅*d* ^−1^) of juvenile walleye pollock interpolated over the range of observed temperatures and prey energy density values across both 2005 and 2010, providing a continuous scale of growth over a broad range of possible environmental and biological scenarios. The observed fish energy density was higher in 2010 (*v_2010_* = 5.29 kJ⋅g^−1^; used in plot shown); therefore this interpolation demonstrates the range of predicted growth for fish with high energy density. Temperatures included 0–16°C to show possible range under variable climate conditions. The dashed rectangle encompasses the range of temperatures and prey energy density values observed in 2005; solid rectangle encompasses values in 2010. Points are shown for average temperature and prey energy density conditions in 2005 and 2010. Predicted growth above 15°C was not possible (black) because the bioenergetics model has a temperature threshold of 15°C.

Predicted maximum growth potential generally increases with temperature and prey energy until temperature-dependent controls limit growth (Figure 3). Predicted growth is negative when available prey energy cannot meet metabolic demands under increased temperatures. Water temperatures were warmer in 2005, therefore juvenile pollock experienced conditions at or near their metabolic threshold at some stations. Colder water temperatures and higher available prey energy in 2010 resulted in better growing conditions over the shelf.

Increasing fish starting weight resulted in lower predicted growth rates in both years because larger fish have higher metabolic demands (Table 3). Increasing fish energy density had a variable effect across stations in 2005 (not shown). In general, the effect of varying fish energy is dependent on initial fish energy and the relative available prey energy at each station. In 2010, increasing fish energy density resulted in lower predicted growth rates across stations when available prey energy was held constant.

Variability in fish starting weight resulted in a broader distribution of predicted growth rates (2005: 0.002–0.109; 2010: 0.017–0.170 *g*⋅*g* ^−1^⋅*d* ^−1^) than variability in fish energy (2005: 0.007–0.013; 2010: 0.022–0.036 *g*⋅*g* ^−1^⋅*d* ^−1^) from Monte Carlo simulations, indicating that the model was more sensitive to inputs of fish weight. The simulated mean predicted growth rates, when varying fish starting weight or fish energy, were lower and less variable for 2005 (0.012±0.009 [mean ± SD] for varying *W*; 0.010±0.001 for varying fish energy) than for 2010 (0.029±0.012 for varying *W*; 0.027±0.002 for varying fish energy).

### Mechanistic Individual-based Model

Predicted mean growth rates from the IBM were 30% (2005) and 46% (2010) lower than maximum growth potential from the bioenergetics model (Tables 3 and 4) as foraging rates are restricted in the IBM based on stomach fullness and the prey selection module (i.e., capture success). The reduction in growth was greater in 2010, resulting in similar predicted growth rates from the IBM in 2005 and 2010. In addition, predicted growth rates from the IBM have a narrower range than maximum growth potential from the bioenergetics model.

**Table 4:** Summary of sensitivity analyses for the IBM model in 2005 and 2010 showing the minimum (min), mean, and maximum (max) growth potential and depth (m) over all stations.

		2005			2010		
Parameter		min	mean	max	min	mean	max
Base	Growth	0.0062	0.0102	0.0121	0.0055	0.0092	0.0123
	Depth	10	44.2	80.9	15	47.4	93
*W* (2.0 g)	Growth	0.004	0.0184	0.0512	0.002	0.0068	0.0254
	Depth	−30.5	−2.6	0.14	−43.3	−2.4	21.7
Prey distribution (Uniform)	Growth	−0.0009	0.005	0.0058	−0.0034	0.001	0.0064
	Depth	−21.4	2.1	15.8	−42.9	−1.8	35.2

Base values are predicted growth (*g*⋅*g* ^−1^⋅*d* ^−1^) and depth (m) of juvenile pollock from the base model scenarios (*W* = 2.5 g, zooplankton prey distributed according to vertical profiles). All other values are predicted changes in growth and depth. Negative changes in depth indicate a shallower distribution; positive values indicate a deeper distribution. Weight is a constant value applied across all station, so varying the parameter acts as a scalar and results in similar spatial patterns across the area. The effect of applying a uniform distribution of zooplankton prey with depth varies across stations.

**Figure 4:**
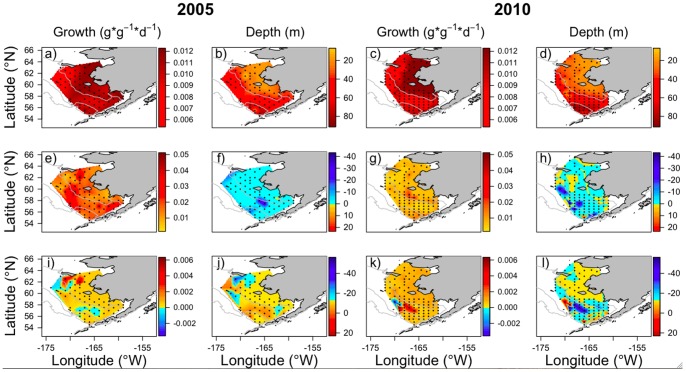
Predicted growth (*g*⋅*g* ^−1^⋅*d* ^−1^) and average depth (m) of juvenile walleye pollock from the IBM. Top panel shows growth (a and c) and average depth (b and d) under the base model scenarios for 2005 and 2010 (*W* = 2.5 g, zooplankton prey distributed according to vertical profiles). Middle panel shows changes in predicted growth (e and g) and average depth (f and h) for 2.0 g fish, highlighting the relative importance of fish size (relative to 2.5 g) and water temperature between years. Lower panel shows changes in predicted growth (i and k) and average depth (j and l) when uniform vertical distributions of prey are implemented, highlighting the effect of zooplankton diel vertical distribution and migrations on juvenile walleye pollock prey selection. Negative changes in depth indicate a shallower distribution; positive values indicate a deeper distribution.

In 2005, growth was positive across the region with moderate growth predicted across the southern shelf. North of 60°N, predicted growth rates decreased from the inner to outer domain (Figure 4a). The average depth (m) of juvenile pollock was 44 m (Table 4), with shallower distributions in the northeast region and deeper distributions in the southern region of the outer domain (Figure 4b). In 2010, growth was positive across the region, with highest predicted growth in the inner domain and areas of lower growth in the middle domain (Figure 4c). The spatial patterns of average depth of juvenile pollock (Figure 4d) mirrored those of 2005 with a slightly deeper average depth of 47 m (Table 4).

#### IBM sensitivity analyses

The effect of smaller fish starting weights on predicted growth was positive across the region, with stronger effects in 2005 than 2010 (Table 4). Similarly, effect strengths varied spatially in both years with areas of higher predicted growth in the middle domain (Figure 4, e and g). In 2005, smaller starting fish weights resulted in shallower depth distributions across the region (mean = −2.6 m; Table 4), with much shallower depths at two stations in the middle domain (Figure 4f). The average change in depth distribution was similar in 2010 (mean = −2.4 m; Table 4), but spatially more variable than in 2005 (Figure 4h).

Applying uniform vertical distributions to prey taxa had variable effects on predicted growth rates in both years, with similarly small effect strengths (Table 4). Under uniform prey distributions, modeled fish may move vertically in response to other cues (i.e., predation risk, thermal boundaries) regardless of diel patterns. In 2005, uniform distributions resulted in increased predicted growth rates at several stations in the northern-most region of the shelf (Figure 4i). While the average depth of juvenile pollock was 2.1 m deeper across the region, fish at some of the northern-most stations had shallower depths (Figure 4j). In 2010, strongest effects on growth were observed in the middle domain of the southern shelf, with high spatial variability (Figure 4k). Changes in the depth of fish in response to uniform prey distributions mirrored spatial patterns in growth effects; stations showing deeper mean depths also resulted in a decrease in growth and vice versa (Figure 4l).

### Spatial Comparison of Bioenergetics- and IBM-predicted Growth

**Figure 5:**
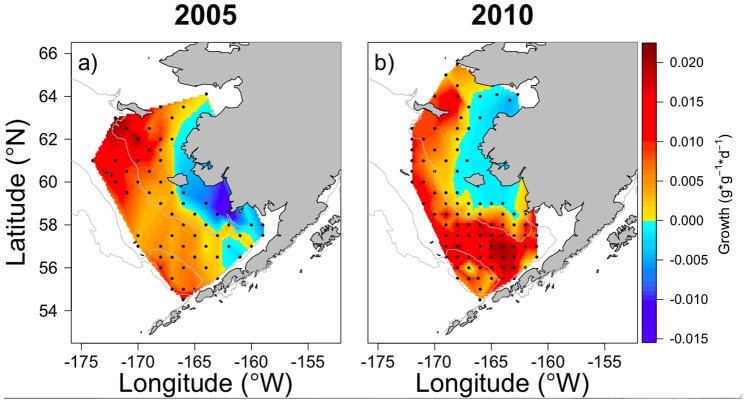
Difference in predicted growth (*g*⋅*g* ^−1^⋅*d* ^−1^) of juvenile walleye pollock between the bioenergetics model and the IBM for 2005 (a) and 2010 (b). Areas of positive differences indicate where maximum growth potential from the bioenergetics model was higher than predicted growth from the IBM.

Predicted growth rates from the IBM were within the range of maximum growth potential from the bioenergetics model, but spatial patterns varied due to differences in input parameters of each model. In both years, the bioenergetics model predicted higher growth rates than the IBM over the middle and outer domains. The greatest difference occurred in the northwest region of the shelf in 2005 (Figure 5a) and over the southern region of the middle domain in 2010 (Figure 5b). The IBM predicted higher growth in the shallow, well-mixed inner domain in both years.

## Discussion

This study demonstrates that warm and cold conditions in the EBS lead to spatial differences in zooplankton species composition, energy content, and abundance, which subsequently affect the feeding ecology and growth of juvenile pollock. Particularly, prey distribution and quality in combination with water temperatures create spatial patterns of increased growth potential (‘hot spots’) that vary with climate conditions. Spatial heterogeneity in growth conditions results from a combination of prey quality and quantity, water temperature, and metabolic costs, which may contribute to size-dependent fish survival and subsequent annual variability in recruitment. We provide evidence that a spatial mismatch between juvenile pollock and growth ‘hot spots’ in 2005 is the mechanism that contributed to poor recruitment to age-1 while a higher degree of overlap in 2010 resulted in 42% greater [32] recruitment to age-1.

**Figure 6:**
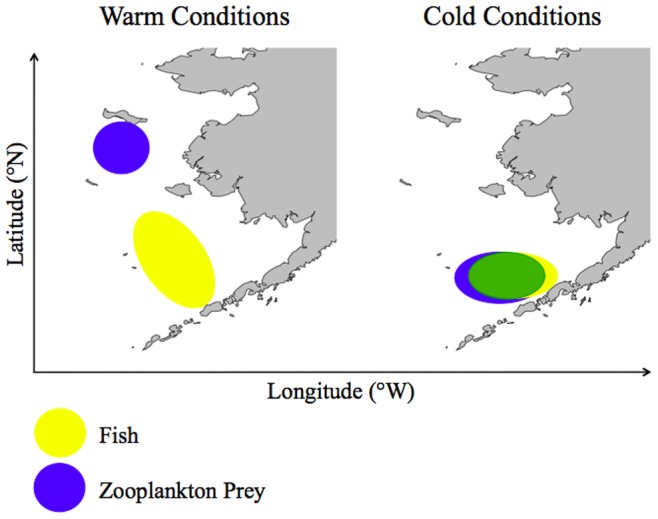
Conceptual figure of the spatial relationship between juvenile fish abundance (yellow) and zooplankton prey availability (blue). Where these areas overlap (green), juvenile fish are predicted to have higher growth rates and increased survival. Under warm climate conditions, there is reduced spatial overlap between juvenile fish and prey availability, resulting in lower overwinter survival and recruitment success to age-1. In colder conditions, increased spatial overlap between juvenile fish and prey availability results in increased overwinter survival and recruitment to age-1.

In the EBS, changes in oceanographic conditions can impact larval and juvenile fish distributions through front formation [33] and subsequent changes in drift trajectories [34]. The resultant variability in fish distributions relative to their prey during late summer and fall may be particularly important because the time period after the completion of larval development and before the onset of winter has been identified as a critical period for energy storage in juvenile pollock [22]. As the spatial distribution of fish, including spawning locations of adult walleye pollock, and zooplankton vary under alternate climate conditions, so do patterns in juvenile fish growth and recruitment success (Figure 6). Here, we find support for the argument that warm years produce smaller, less energy-rich prey and that this reduced prey quality, in combination with higher metabolic demands, results in lower growth of juvenile pollock. Conversely, cold years produce larger, more energy-rich prey which, when combined with lower metabolic demands, are favorable for juvenile pollock growth and survival. Thus, mechanisms responsible for controlling growing conditions during the critical pre-winter period can be linked to variability in recruitment.

Projected declines in walleye pollock recruitment under changing climate conditions [11] do not account for adaptive behaviors or changes to phenology that could enable fish to maintain higher growth rates. The sensitivity analyses helped to identify when and where favorable growth conditions may occur under alternate climate conditions. In the bioenergetics model, varying fish size had a stronger effect on growth potential than changes in initial fish energy density. Larger fish have greater capacity for growth due to increased gape size, which allows them to take advantage of larger, more energy rich prey resources (e.g., euphausiids) prior to winter. The sensitivity analysis of increasing water temperatures showed weaker effects in the cold year of 2010 because fish had a broader range of temperatures over which growth potential was relatively high (Figure 3), including warmer surface waters and a colder refuge in deeper waters that allows fish to conserve energy and avoid predation. In 2005, fish were near thermal limits based on temperature-dependent functions in the bioenergetics model; hence further increases in temperature are predicted to result in negative growth. Increasing available prey energy also had a stronger effect in the warm year of 2005 because metabolic demands were greater and mean prey energy density was lower than in 2010.

The relative foraging rate was held constant at η = 1 across all bioenergetics model scenarios, but lower values would better reflect realistic foraging rates and could exacerbate thermal constraints on growth. To maintain positive growth rates at half of all the stations required relative foraging rates of η = 0.71 in 2005 and η = 0.57 in 2010. These values correspond to a 29% and 43% reduction in achieved growth relative to maximum growth potential and are similar to the mean differences between growth rates in the bioenergetics and IBM models (i.e., 30% in 2005 and 46% in 2010), providing support of model agreement. A higher relative foraging rate was required in 2005 in order to achieve positive growth at half of all stations, similar to results based on larger juvenile and adult walleye pollock [25], indicating that juvenile pollock growth was more prey limited and constrained by temperature in 2005 than in 2010. Thus, a greater reduction in both achieved growth from the IBM relative to maximum growth potential and relative foraging rates was observed in 2010 compared to 2005. In 2010, zooplankton abundance was lowest in areas with higher concentrations of juvenile pollock, potentially indicating prey limitation. Our study was not designed to explicitly test this question; other research indicates local depletion of euphausiids by age-1 and older pollock is possible [35], but shelf-wide euphausiid abundance is probably not controlled by pollock predation (i.e., top-down control; P. Ressler, pers. comm.).

The vertical behavior of modeled juvenile pollock in the IBM moderated predicted growth rates leading to differences across domains based on stratification. Smaller (i.e., younger) fish were predicted to move shallower in the water column to improve prey detection, which is dependent on eye development and light availability. Moving into the surface layer also exposed juvenile pollock to higher predation risk because of the stronger light intensity. In the middle and outer domains, once sufficient growth was attained, fish were predicted to move deeper to seek refuge from predation. While the models were run at all stations in both years, observed juvenile pollock abundances were concentrated over the middle and outer domains in 2005 and over small regions of the southern shelf and outer domain in 2010. Few fish were observed in the well-mixed inner domain, possibly due to reduced growth potential based on available prey energy or lack of stratification and predation refuge in deeper waters. Additionally, the inner front, which delineates the stratified middle domain from the well-mixed inner domain [33], may act as a barrier to juvenile pollock distribution [36].

Spatial patterns in juvenile pollock growth differed between models; these differences elucidate underlying mechanisms in feeding potential and ultimately the possible causes for growth ‘hot spots’ and variability in recruitment success between warm and cold climate conditions. The bioenergetics model uses biomass-weighted mean energy density of available prey, assuming fish feed proportional to what is available in the environment. The IBM is length-based and growth is dependent on available prey resources, light conditions, metabolism, development of the fish, and fish behavior. In the middle and outer domains where the water column is stratified, the bioenergetics model predicted higher growth than the IBM; the bioenergetics model allowed fish to feed at maximum consumption while the IBM indicated that fish moved deeper in the water column to conserve energy or avoid predation. In the inner domain, the IBM predicted higher growth; here juvenile pollock may opt to take advantage of available prey and warmer water temperatures to maximize growth because predator avoidance in deeper waters was not an option.

Comparing the bioenergetics model and the IBM provided insights that could not be gained by either approach alone. For example, the bioenergetics model highlights the importance of differences in prey energy, a metric not included in the IBM, in determining spatial patterns of growth. On the other hand, the mechanistic feeding behavior implemented in the IBM highlights the role of prey size composition, the vertical distribution of prey, and the tradeoff between predator avoidance and maximizing growth. In practice, data requirements may limit the applicability of the IBM, whereas the bioenergetics model can be applied when less information on prey resources is available. Future research could benefit from including information on prey energy into IBMs to disentangle not only the importance of species composition, size composition, spatial distribution and abundance of prey, but also the importance of prey quality.

Warm temperature conditions are predicted to result in reduced prey quality and low energy density of juvenile pollock in late summer [9], [13]. Warmer water temperatures are associated with decreased growth [this study], resulting in lower overwinter survival and recruitment to age-1 [32]. The warm years of 2002–2005 had 67% lower average recruitment to age-1 relative to the cold years of 2008–2010, although variability during the cold years was very high with strong year classes in 2008 and 2010 separated by a weak 2009 cohort [32]. These findings agree with projected declines in recruitment of age-1 walleye pollock [11] under increased summer sea surface temperatures of 2°C predicted by 2050 [37]. Our results corroborate these previous studies and suggest that climate-driven increases in water temperature will have the largest effect on recruitment during anomalously warm years.

This study provides evidence that climate-driven changes in prey dynamics can have ecosystem-level consequences via bottom-up control of fish populations in sub-arctic marine ecosystems. This work has improved our understanding of the mechanisms behind recruitment variability, in particular the underlying spatial patterns that drive relationships between prey availability, water temperature, growth, and survival. Our findings inform ongoing discussions of climate effects on predator-prey interactions and recruitment success of marine fishes.

## Supporting Information

Figure S1 **Eastern Bering Sea with locations of sampling stations at which the bioenergetics model and IBM were run in 2005 (•) and 2010 (□).** The Monte Carlo Station (▴) is the representative station used for Monte Carlo simulations. Depth contours are shown for the 50 m, 100 m, and 200 m isobaths.
(TIF)

Figure S2 **Water temperatures interpolated across all stations (•) sampled by the CTD.** Top panel shows the mean temperature in the upper 30 m of the water column in 2005 (a) and 2010 (b). Bottom panel shows the mean temperature below 40 m in 2005 (c) and 2010 (d).
(TIF)

Table S1 **Stage, sampling gear, length range, width, biomass (g, wet weight), and energy density (kJ⋅g^−1^, wet weight) values for the main prey items of juvenile walleye pollock in late summer 2005 and 2010.** Biomass estimates were obtained during processing of the zooplankton samples from 2005 (warm) and 2010 (cold) (NA = stage was not collected); energy density values were obtained from zooplankton collected in the eastern Bering Sea during 2004 (warm) and 2010 (cold). Single estimates of energy density (shown in bold) were used when year-specific information was not available for individual taxa. Stage abbreviations as follows: A = adult, AF = adult female, AM = adult male, C = copepodite, XS = extra small, S = small, M = medium, L = large, J = juvenile.
(DOCX)

Table S2 **Component equations of the bioenergetics model used to estimate maximum growth potential (** ***g*** **⋅** ***g*** **^−1^⋅** ***d*** **^−1^) of juvenile walleye pollock.**
(DOCX)
