# Supplementary figures and images for: Spatial Match-Mismatch between Juvenile Fish and Prey Provides a Mechanism for Recruitment Variability across Contrasting Climate Conditions in the Eastern Bering Sea
Source: PLoS One. 2013 Dec 31;8(12):e84526. doi: 10.1371/journal.pone.0084526 (PMC3877275; doi:10.1371/journal.pone.0084526)

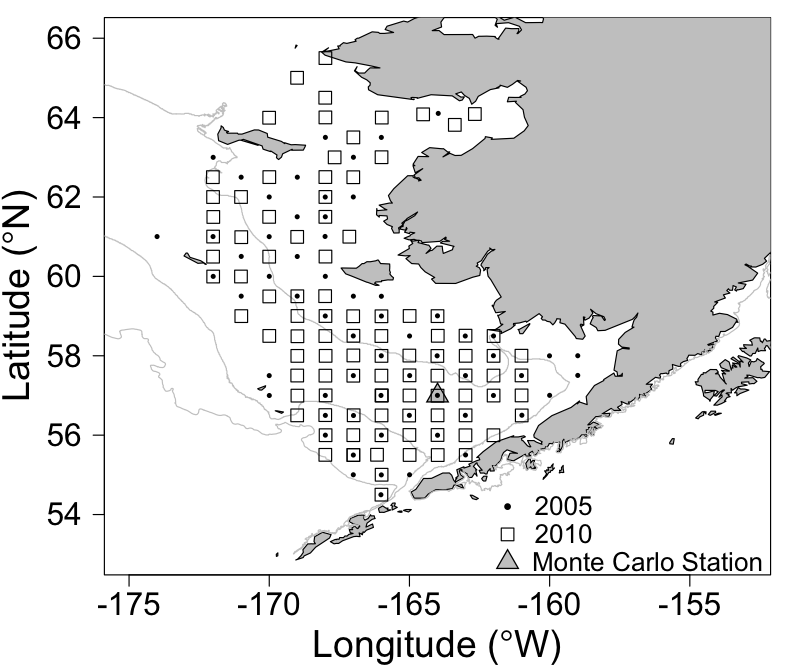

Supplement: Figure S1 — Eastern Bering Sea with locations of sampling stations at which the bioenergetics model and IBM were run in 2005 (•) and 2010 (□). The Monte Carlo Station (▴) is the representative station used for Monte Carlo simulations. Depth contours are shown for the 50 m, 100 m, and 200 m isobaths. (TIF) [file pone.0084526.s001.tif]

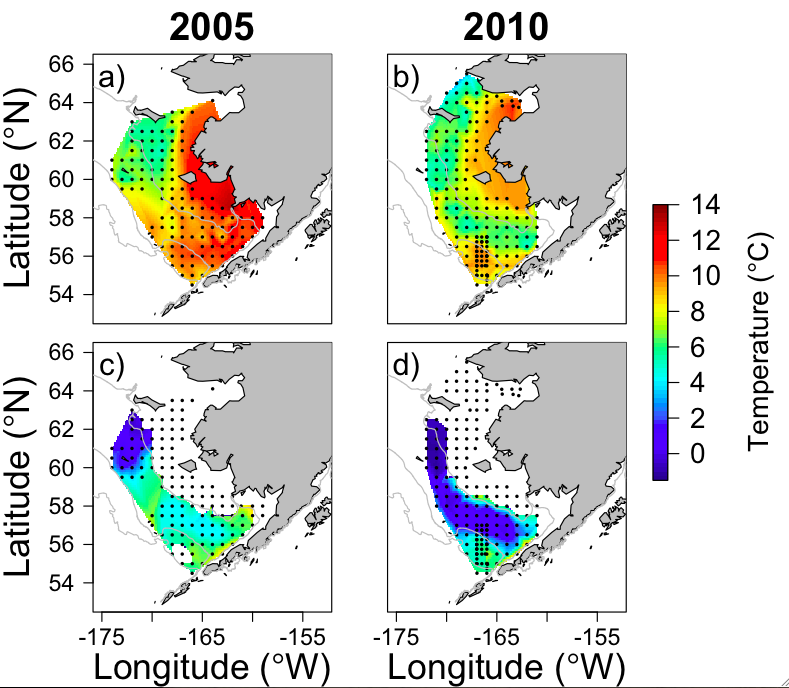

Supplement: Figure S2 — Water temperatures interpolated across all stations (•) sampled by the CTD. Top panel shows the mean temperature in the upper 30 m of the water column in 2005 (a) and 2010 (b). Bottom panel shows the mean temperature below 40 m in 2005 (c) and 2010 (d). (TIF) [file pone.0084526.s002.tif]

| Consumption |  |
| --- | --- |
|  |  |
|  |  |
|  |  |
|  |  |
|  |  |
| Respiration |  |
|  |  |
|  |  |
|  |  |
|  |  |
|  |  |
| Egestion |  |
| Excretion | 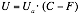 |

Supplement: Table S2 — Component equations of the bioenergetics model used to estimate maximum growth potential ( g ⋅ g −1⋅ d −1) of juvenile walleye pollock. (DOCX) [file pone.0084526.s004.docx]
