# Supplementary material for: Spatial Match-Mismatch between Juvenile Fish and Prey Provides a Mechanism for Recruitment Variability across Contrasting Climate Conditions in the Eastern Bering Sea
Source: PLoS One. 2013 Dec 31;8(12):e84526. doi: 10.1371/journal.pone.0084526 (PMC3877275; doi:10.1371/journal.pone.0084526)
Supplement: Table S1 — Stage, sampling gear, length range, width, biomass (g, wet weight), and energy density (kJ⋅g−1, wet weight) values for the main prey items of juvenile walleye pollock in late summer 2005 and 2010. Biomass estimates were obtained during processing of the zooplankton samples from 2005 (warm) and 2010 (cold) (NA = stage was not collected); energy density values were obtained from zooplankton collected in the eastern Bering Sea during 2004 (warm) and 2010 (cold). Single estimates of energy density (shown in bold) were used when year-specific information was not available for individual taxa. Stage abbreviations as follows: A = adult, AF = adult female, AM = adult male, C = copepodite, XS = extra small, S = small, M = medium, L = large, J = juvenile. (DOCX) [file pone.0084526.s003.docx]

| **Species** | **Stage** | **Gear** | **Length range (mm TL)** | **Width (mm)** | **Warm biomass (g WW)** | **Cold biomass (g WW)** | **Warm energy density (kJ•g^-1^ WW)** | **Cold energy density (kJ•g^-1^ WW)** | **Comments** |
| --- | --- | --- | --- | --- | --- | --- | --- | --- | --- |
| *Acartia clausi* | A | Juday | 0.25 – 1.4*^a^* | 0.22*^b^* | 3.5 E-05 | 3.5 E-05 | **3.816***^c^* | **3.816** *^c^* |  |
|  | AF | Juday | 0.8 – 1.4 *^a^* | 0.29 *^b^* | NA | 4.5 E-05 |  |  |  |
|  | AM | Juday | 0.8 – 1.2 *^a^* | 0.27 *^b^* | NA | 2.5 E-05 |  |  |  |
| *Acartia* sp. | A | Juday | 0.25 – 0.93 *^a^* | 0.16 *^b^* | 1.85 E-05 | 1.9 E-05 |  |  |  |
|  | I | Juday | 0.25 – 0.42 *^a^* | 0.09 *^b^* | NA | 4.1 E-06 |  |  | Length range for *A. clausi* |
|  | II | Juday | 0.42 – 0.51 *^a^* | 0.12 *^b^* | NA | 9.4 E-06 |  |  | Length range for *A. clausi* |
|  | III | Juday | 0.51 – 0.65 *^a^* | 0.16 *^b^* | NA | 1.7 E-05 |  |  | Length range for *A. clausi* |
|  | IV | Juday | 0.65 – 0.76 *^a^* | 0.19 *^b^* | 1.0 E-05 | 2.5 E-05 |  |  | Length range for *A. clausi* |
|  | V | Juday | 0.76 – 0.93 *^a^* | 0.23 *^b^* | 2.7 E-05 | 4.0E-05 |  |  | Length range for *A. clausi* |
| *Calanus marshallae* | AF | Bongo | 3.2 – 4.2 *^a^* | 0.78*^d^* | 0.003 | 0.002 | 5.325*^f^* | 5.732*^g^* |  |
|  | AM | Bongo | 3.5 – 4 *^a^* | 1.0*^e^* | 0.0026 | 0.0017 |  |  |  |
|  | I | Bongo | 0.5 – 0.7 *^a^* | 0.16 *^e^* | 5.25 E-05 | 5.25 E-05 |  |  |  |
|  | II | Bongo | 1.2 – 1.5 *^a^* | 0.36 *^e^* | 9.19 E-05 | 1.33 E-04 |  |  |  |
|  | III | Bongo | 1.6 – 2.3 *^a^* | 0.68 *^d^* | 1.9 E-04 | 2.7 E-04 |  |  |  |
|  | I-III | Bongo | 0.5 – 2.3 *^a^* | 0.37 *^e^* | 1.13 E-04 | 2.2 E-04 |  |  |  |
|  | IV | Bongo | 2.3 – 2.6 *^a^* | 0.69 *^d^* | 5.82 E-04 | 5.24 E-04 |  |  |  |
|  | V | Bongo | 2.8 – 3.8 *^a^* | 0.73 *^d^* | 0.002 | 0.0016 |  |  |  |
| *Centropages abdominalis* | A | Juday | 0.3 – 2.1 *^a^* | 0.36*^h^* | 7.49 E-05 | 5.73 E-05 | **3.843***^i^* | **3.843** *^i^* |  |
|  | AF | Juday | 1.6 – 2.1 *^a^* | 0.53 *^h^* | 1.61 E-4 | 1.36 E-4 |  |  |  |
|  | AM | Juday | 1.5 *^a^* | 0.5 *^h^* | 9.14 E-05 | 1.16 E-4 |  |  |  |
|  | C | Juday | 1.13 *^a^* | 0.5 *^h^* | 2.33 E-05 | 2.33 E-05 |  |  |  |
|  | I | Juday | 0.3 *^a^* | 0.16 *^h^* | NA | 2.92 E-05 |  |  |  |
|  | II | Juday | 0.38 *^a^* | 0.22 *^h^* | NA | 1.5 E-05 |  |  |  |
|  | III | Juday | 0.5 *^a^* | 0.29 *^h^* | 1.32 E-05 | 2.82 E-05 |  |  |  |
|  | I-III | Juday | 0.33 *^a^* | 0.17 *^h^* | NA | 9.1 E-06 |  |  |  |
|  | IV | Juday | 0.65 *^a^* | 0.39 *^h^* | NA | 4.25 E-05 |  |  |  |
|  | V | Juday | 0.85 *^a^* | 0.5 *^h^* | 8.55 E-05 | 6.89 E-05 |  |  |  |
| *Eucalanus bungii* | A | Bongo | 4.8 – 8 *^a^* | 1.7*^e^* | 0.0023 | 8.67 E-05 | 3.916*^j^* | 4.194*^k^* |  |
|  | AF | Bongo | 6 – 8 *^a^* | 1.86 *^e^* | 0.0086 | 0.0086 |  |  |  |
|  | AM | Bongo | 4.8 – 5.5 *^a^* | 1.37 *^e^* | 0.0031 | 0.0041 |  |  |  |
|  | I | Bongo | 1.3 – 1.6 *^a^* | 0.39 *^e^* | 4.5 E-05 | 5.3 E-05 |  |  |  |
|  | II | Bongo | 2 – 2.2 *^a^* | 0.56 *^e^* | 1.96 E-4 | 1.56 E-4 |  |  |  |
|  | III | Bongo | 2.9 – 3 *^a^* | 0.79 *^e^* | 4.92 E-4 | 2.66 E-4 |  |  |  |
|  | I-III | Bongo | 1.3 – 3 *^a^* | 0.57 *^e^* | 2.44 E-04 | 8.41 E-4 |  |  |  |
|  | IV | Bongo | 3.36 – 3.8 *^a^* | 0.95 *^e^* | 0.0011 | 0.0009 |  |  |  |
|  | V | Bongo | 4.5 – 5.2 *^a^* | 1.29 *^e^* | 0.0027 | 0.0034 |  |  |  |
| *Limacina helicina* | XS | Bongo | 0.1 – 0.5*^l^* | 0.3 *^l^* | NA | 6.93 E-05 | 2.51*^m^* | 2.766*^g^* |  |
|  | S | Bongo | 0.5 – 2 *^l^* | 1.25 *^l^* | 9.40 E-05 | 1.58 E-4 |  |  |  |
|  | M | Bongo | 2 – 4 *^l^* | 3 *^l^* | 3.71 E-4 | 8.29 E-4 |  |  |  |
|  | L | Bongo | 4 - 10 *^l^* | 7 *^l^* | 0.0026 | 0.0045 |  |  |  |
| *Neocalanus cristatus* | AF | Bongo | 8.5 – 10.4*^a^* | 2.5 *^e^* | NA | 0.0137 | 3.253*^n^* | 3.39*^g^* |  |
|  | III | Bongo | 3.2 *^a^* | 0.85 *^e^* | 8.83 E-4 | 0.0013 |  |  |  |
|  | IV | Bongo | 4.9 – 5.3 *^a^* | 1.36 *^e^* | 0.0059 | 0.0025 |  |  |  |
|  | V | Bongo | 7.1 – 8.9 *^a^* | 2.13 *^e^* | 0.019 | 0.015 |  |  |  |
| *N. plumchrus* | V | Bongo | 4.1 – 5.2 *^a^* | 1.24 *^e^* | 0.00395 | 0.0041 | 4.207*^o^* | 4.676*^g^* |  |
| *Oikopleura* sp. | A | Bongo | 0.1 – 0.6*^p^* | 0.35 *^p^* | 1.73 E-4 | 1.7 E-4 | 4.076*^q^* | 4.025*^r^* |  |
| *Pseudocalanus* spp. | A | Juday | 0.65 – 1.2*^s^* | 0.29 *^d^* | 4.44 E-05 | 3.6 E-05 | **3.951***^t^* | **3.951** *^t^* | Length range for *P. moultoni* |
|  | AF | Juday | 1.05 – 2.27 *^s^* | 0.42 *^d^* | 8.27 E-05 | 8.01 E-05 |  |  |  |
|  | AM | Juday | 0.91 – 1.74 *^s^* | 0.35*^b^* | 4.13 E-05 | 5.42 E-05 |  |  |  |
|  | I | Juday | 0.5 – 0.7 *^s^* | 0.16 *^b^* | NA | 5.98 E-06 |  |  |  |
|  | II | Juday | 0.65 – 0.8 *^s^* | 0.19 *^b^* | 1.01 E-05 | 1.08 E-05 |  |  |  |
|  | III | Juday | 0.8 – 1 *^s^* | 0.24 *^b^* | 1.26 E-05 | 2.0 E-05 |  |  |  |
|  | I-III | Juday | 0.5 – 1 *^s^* | 0.2 *^b^* | NA | 1.01 E-05 |  |  |  |
|  | IV | Juday | 1 – 1.2 *^s^* | 0.3 *^d^* | 3.06 E-05 | 3.19 E-05 |  |  |  |
|  | V | Juday | 1.2 – 1.5 *^s^* | 0.37 *^d^* | 8.89 E-05 | 4.93 E-05 |  |  |  |
|  | II-V | Juday | 0.65 – 1.5 *^s^* | 0.29 *^d^* | 3.55 E-05 | 2.8 E-05 |  |  |  |
| *Thysanoessa inermis* | A | Bongo | 10.1 – 29.2*^u^* | 2.4 *^d^* | NA | 0.083 | **4.99***^m^* | **4.99** *^m^* |  |
|  | AF | Bongo | 10.1 – 29.2 *^u^* | 2.4 *^d^* |  | 0.10 |  |  |  |
|  | AM | Bongo | 10.1 – 29.2 *^u^* | 2.4 *^d^* | 0.069 | NA |  |  |  |
|  | J | Bongo | 8.5 – 13.8 *^u^* | 1.4 *^d^* | NA | 0.011 |  |  |  |
|  | J (L) | Bongo | 11.1 – 13.8 *^u^* | 1.5 *^d^* | 0.061 | 0.11 |  |  |  |
|  | J (S) | Bongo | 8.5 – 11.1 *^u^* | 1.2 *^d^* | 0.011 | 0.024 |  |  |  |
| *T. inspinata* | J | Bongo | 12 – 17*^v^* | 2.2*^w^* | 0.0012 | 0.0128 | **4.99***^x^* | **4.99***^x^* |  |
| *T. raschii* | A | Bongo | 7 – 29.1*^y^* | 3.3 *^d^* | 0.006 | NA | 4.308*^aa^* | 5.231*^g^* |  |
|  | AF | Bongo | 7 – 29.1 *^y^* | 3.3 *^d^* | 0.077 | 0.0903 |  |  |  |
|  | AM | Bongo | 15.3 – 20.2*^z^* | 2.7 *^d^* | 0.046 | 0.088 |  |  |  |
|  | J | Bongo | 7.4 – 8.4 *^z^* | 1.45 *^d^* | 0.005 | 0.0117 |  |  |  |
|  | J (L) | Bongo | 7.9 – 8.4 *^z^* | 1.5 *^d^* | 0.0476 | 0.0936 |  |  |  |
|  | J (S) | Bongo | 7.4 – 7.9 *^z^* | 1.4 *^d^* | 0.0089 | 0.0059 |  |  |  |

*^a^* [1]; *^b^* Estimated width = 26.7% of length (based on *Pseudocalanus* sp. relationship); *^c^* Energy density estimated from % lipid (2.25% wet weight assuming 80% moisture [2]) using the regression relationship: ED = (0.4098•% lipid) + 19.287; *^d^* E. Fergusson, NOAA/AFSC, unpublished data; *^e^* Estimated width = 26.6% of length (based on *C. marshallae* relationship); *^f^* Energy density estimated from % lipid (10.5%; R. Heintz, NOAA/AFSC, unpublished data); *^g^* R. Heintz, NOAA/AFSC, unpublished data; *^h^* [3]; *^i^* Energy density estimated from % lipid (2.6% wet weight [4]); *^j^* Energy density estimated from % lipid (3.55%; R. Heintz, NOAA/AFSC, unpublished data); *^k^* Energy density estimated as 7.1% higher in cold years (based on copepod data; [this study]); *^l^* C. Stark, UAF, unpublished data; *^m^* 2006 collection (R. Heintz, NOAA/AFSC, unpublished data); *^n^* Energy density estimated from % lipid (5.85%; R. Heintz, NOAA/AFSC, unpublished data); *^o^* Energy density estimated from % lipid (6.83%; R. Heintz, NOAA/AFSC, unpublished data); *^p^* Trunk length/width [5]; *^q^* Energy density estimated from % lipid of Chaetognatha (2.67%; R. Heintz, NOAA/AFSC, unpublished data); *^r^* Energy density estimated from % lipid of Chaetognatha (2.04%; R. Heintz, NOAA/AFSC, unpublished data); *^s^* [6]; *^t^* Energy density estimated from % lipid (4% wet weight [7]); *^u^* Carapace width from [8]; converted to TL using equations from [9]; *^v^* Length range of ‘spineless’ *T. longipes* [10]; *^w^* Estimated width as 15% of length; *^x^* Used energy density of *T. inermis*; *^y^* Minimum size for *T. inermis* and maximum size for *T. spinifera* [8]; converted to TL using equations from [9]; *^z^* Minimum size for *T. inermis* and maximum size for *T. spinifera* [11]; converted to TL using equations from [9]; *^aa^* Energy density estimated as 17.65% higher in cold years (R. Heintz, NOAA/AFSC, unpublished data).

**References**

1. Gardner GA, Szabo I (1982) British Columbia pelagic marine copepoda: an identification manual and annotated bibliography. Can Spec Pub Fish Aquat Sci 62. 536 p.

2. Yamamoto T, Teruya K, Hara T, Hokazono H, Hashimoto H, et al. (2008) Nutritional evaluation of live food organisms and commercial dry feeds used for seed production of amberjack *Seriola dumerili*. Fish Sci 74: 1096-1108.

3. Lough RG, Buckley LJ, Werner FE, Quinlan JA, Edwards KP (2005) A general biophysical model of larval cod (*Gadus morhua*) growth applied to populations on Georges Bank. Fisheries Oceanography 14: 241-262.

4. Lee RF, Hagen W, Kattner G (2006) Lipid storage in marine zooplankton. Mar Ecol Prog Ser 307: 273-306.

5. Tomita M, Ikeda T, Shiga N (1999) Production of *Oikopleura longicauda* (Tunicata: Appendicularia) in Toyama Bay, southern Japan Sea. J Plankton Res 21(12): 2421-2430.

6. Frost BW (1989) A taxonomy of the marine calanoid copepod genus *Pseudocalanus*. Can J Zool 67: 525-551.

7. Peters J (2006) Lipids in key copepod species of the Baltic Sea and North Sea – implications for life cycles, trophodynamics and food quality. PhD Dissertation. University of Bremen. 177 p.

8. Pinchuk AI, Coyle KO (2008) Distribution, egg production and growth of euphausiids in the vicinity of the Pribilof Islands, southeastern Bering Sea, August 2004. Deep Sea Res Part II Top Stud Oceanogr 55: 1792-1800.

9. Pinchuk AI, Hopcroft RR (2007) Seasonal variations in the growth rates of euphausiids (*Thysanoessa inermis*, *T. spinifera*, and *Euphausia pacifica*) from the northern Gulf of Alaska. Mar Biol 151: 257-269.

10. Kathman RD, Austin WC, Saltman JC, Fulton JD (1986) Identification manual to the Mysidacea and Euphausiacea of the northeast Pacific. Can Spec Pub Fish Aquat Sci 93. 411 p.

11. Falk-Petersen S (1985) Growth of the euphausiids *Thysanoessa inermis*, *Thysanoessa raschii*, and *Meganyctiphanes norvegica* in a subarctic fjord, North Conway. Can J Fish Aquat Sci 42: 14-22.
